# Early assessment of physical capacity and pain is associated with 1-month response following epidural steroid injection in patients with sciatica due to degenerative lumbar disorders

**DOI:** 10.1016/j.bas.2026.106111

**Published:** 2026-05-29

**Authors:** Michal Ziga, Martin N. Stienen, Anna Maria Zeitlberger, Ulf C. Schneider, Luca Regli, Victor Staartjes, Nicolai Maldaner

**Affiliations:** a Department of Neurosurgery, Cantonal Hospital Lucerne, Lucerne, Switzerland; b Faculty of Health Sciences and Medicine, University of Lucerne, Lucerne, Switzerland; c Department of Neurosurgery, Cantonal Hospital St. Gallen, H-OCH Health Ostschweiz, Medical School, University of St.Gallen, St. Gallen, Switzerland; d 1st Faculty of Medicine, Charles University in Prague, Prague, Czech Republic; e Department of Neurosurgery, University Hospital Zurich & Clinical Neuroscience Centre, University of Zurich, Zurich, Switzerland; f Department of Clinical Neuroscience, Karolinska Institutet, Stockholm, Sweden; g Capio Spine Center Stockholm, Löwenströmska Hospital, Upplands Väsby, Sweden

**Keywords:** 6-Min walking test, 6WT, Objective functional impairment, Functional self-assessment, Steroid infiltration

## Abstract

**Introduction:**

Epidural steroid injections (ESI) are widely used to treat sciatica from degenerative lumbar disorders (DLD), but objective functional improvement and the predictive value of early response remain unclear.

**Research question:**

Does ESI improve pain and physical capacity in DLD-related sciatica, and can early achievement of the minimum clinically important difference (MCID) predict outcomes at 1 month?

**Methods:**

In this prospective study, 45 patients with DLD-related sciatica were evaluated before and after ESI at 1 day (1D), 1 week (1W), and 1 month (1M). Physical capacity was measured using a smartphone-based 6-min walking test (6WT); pain was assessed with Visual Analogue Scale scores for leg and back pain (VAS Leg, VAS Back). Agreement of MCID classification across follow-ups was analyzed with Cochran's Q and Fleiss' kappa. Sensitivity, specificity, and predictive values evaluated whether early MCID predicted outcomes at 1M.

**Results:**

Forty-five patients (mean age 51 ± 13 years; 53% female) were included. Walking distance increased from 457 m (SD 92; z −1.0 ± 1.1) at baseline to 523 m (SD 71; z −0.5 ± 1.1) at 1D (p < 0.001), with no further change to 1M. MCID classification showed good-to-excellent agreement (Cochran's Q p > 0.05; Fleiss' κ > 0.6). Achieving MCID within 1W predicted MCID at 1M with 100% sensitivity for VAS Leg and 80% for walking distance.

**Discussion and conclusion:**

6WT performed within one week is associated with treatment success at one month, supporting its role as a valuable objective tool in early outcome assessment.

## Highlights

• Early assessment after epidural steroid injection predicts short-term treatment success.
• Objective functional capacity measured by smartphone-based 6-min walking test improves rapidly after ESI.
• Most improvements in pain and walking capacity occur within the first day and plateau thereafter.
• Achieving MCID within one week strongly predicts MCID attainment at one month.
• The smartphone-based 6WT is a practical objective tool to complement PROMs in clinical follow-up.

## Abbreviations

MCID: minimal clinically important difference
PROMs: patient-reported outcome measures
6WT: 6-min walking test
6WD: 6-min walking distance
SD: standard deviation
DLD: degenerative lumbar disorders
ESI: epidural steroid injection
m: meters
VAS: visual analogue scale
ODI: Oswestry Disability Index
app: application
LDH: lumbar disc herniation
GPS: global positioning system
FU: follow-up
TUG: Timed Up and Go
CI: confidence interval

## 1 Introduction

Patients with sciatica secondary to degenerative lumbar disorders (DLD) are frequently confronted with the decision between surgical and conservative treatment options aimed at pain relief and functional improvement. In the absence of severe neurological deficits or intractable pain, conservative management is generally recommended as first-line therapy. These non-surgical approaches are well established, widely applied, and often associated with substantial symptom relief or complete recovery. (Prescher, 1998).^,^ (Gautschi et al., 2014)

Accurate monitoring of symptom progression is essential for guiding therapeutic decision-making. Traditionally, patient-reported outcome measures (PROMs) have been used to assess pain intensity and functional status. (Mannion et al., 2015)^,^ (Mannion et al., 2005) However, the inherently subjective nature of PROMs and their susceptibility to reporting bias have motivated the development of objective functional assessments. (Gautschi et al., 2014)^,^ (Carragee, 2010)^,^ (Tomkins-Lane and Battié, 2013) Objective performance-based tests, such as the 6-min walking test (6WT) and the Timed Up and Go (TUG) test, have emerged as reliable, validated alternatives that are also well accepted by patients (Stienen et al., 2019a, 2019b; Gautschi et al., 2016a; Sosnova et al., 2021).

Among these tools, the 6WT has gained particular relevance, especially in its smartphone-based implementation. The test objectively quantifies functional impairment by measuring the maximum distance a patient can walk within 6 min (6-min walking distance; 6WD). Results can be standardized using age- and sex-adjusted z-scores derived from normative reference populations. (Sosnova et al., 2020)^,^ (Zeitlberger et al., 2020) This objective assessment is particularly valuable in surgical and interventional contexts, where PROMs may reach a ceiling effect during follow-up, limiting their ability to detect further functional improvement (Ziga et al., 2023). In such cases, objective measures like the 6WT provide additional discriminatory power for outcome evaluation.

Previous studies involving both surgically and conservatively treated patients have demonstrated the validity, responsiveness, and clinical utility of the 6WT. (Stienen et al., 2019a)^,^ (Sosnova et al., 2020)^,^ (Maldaner et al., 2020a; Ziga et al., 2024, 2025) However, longitudinal data on postinterventional trajectories and the predictive value of early functional improvement—particularly in patients with sciatica due to DLD undergoing epidural steroid injection (ESI)—remain limited.

The primary aim of this study was therefore to evaluate changes in subjective pain intensity, assessed using the Visual Analogue Scale (VAS), and objective physical capacity, measured with a smartphone-based 6WT, following ESI in patients with sciatica due to DLD. A secondary aim was to determine whether achieving the minimum clinically important difference (MCID) within one week (1W) after ESI predicts MCID attainment at one month (1M).

## 2 Methods

### 2.1 Patient inclusion

Between July 2020 and March 2022, a prospective screening was conducted at the Department of Neurosurgery, Cantonal Hospital St. Gallen, Switzerland. Adult patients diagnosed with sciatica due to DLD—specifically lumbar disc herniation (LDH) or uni- or bilateral lateral recess stenosis—were eligible if they were treated conservatively with ESI in an outpatient setting.

In all patients, ESI consisted of a standardized mixture of a local anesthetic (Rapidocaine 1%, 2 ml) and corticosteroid (Dexamethasone 8 mg, 1 ml). All injections were performed under fluoroscopic guidance in antero-posterior and lateral projection. The route (transforaminal vs. interlaminar) and level was selected based on pathology, its location and laterality of symptomas (unilateral vs. bilateral sciatica). These procedures are standardized across the physicians at the study departement. If applicable, concomitant medications and physiotherapy initiated prior to the ESI were continued throughout the study period without modification.

Eligibility was determined using predefined inclusion and exclusion criteria detailed in Supplemental Digital Content 1. These criteria were strictly applied to ensure a homogeneous study population and to maintain methodological rigor.

### 2.2 The 6WT-App

The 6WT app (Supplemental Fig. 1) is a smartphone application that quantifies the maximum distance walked in 6 min (6WD, meters) using Global Positioning System (GPS) tracking (Stienen et al., 2019a). During the test, elapsed time and distance are continuously displayed. Participants are instructed to walk as fast as possible for the full duration.

Patients with DLD often exhibit pain-related gait impairment, reduced walking speed, and frequent rest periods, all of which contribute to a reduced 6WD. At the initial consultation, patients received standardized instructions on downloading and using the app. To ensure the reproducibility of objective functional assessments, several standardization measures were implemented for the 6WT. Patients were instructed to perform the tests using the same walking route (typically a quiet, level hallway or outdoor path), at a similar time of day, and wearing consistent footwear for each assessment. Additionally, patients were advised to maintain a consistent timing of their analgesic intake relative to the performance of the 6WT at each follow-up interval (1 day, 1 week, and 1 month) to minimize the confounding effect of fluctuating medication levels on physical capacity, in accordance with previously published protocols (Ziga et al., 2024). The application is freely available for both Apple iOS and Android platforms.

### 2.3 Data collection and patient reported outcome measures (PROMs)

Upon study inclusion, demographic and clinical data were prospectively collected. Outcome assessment included both subjective PROMs and objective functional testing with the 6WT. Measurements were obtained at four predefined time points: baseline (prior to ESI), one day (1D), one week (1W), and one month (1M) following ESI.

PROMs included the Visual Analogue Scale (VAS) for leg pain and back pain intensity (ranging from 0 (none) to 10 (severe pain)).

### 2.4 Ethical considerations

The study was approved by the local ethic committee (Kantonale Ethikkommission, EKOS – 2019-01209) and was registered as NCT04062942 at http://clinicaltrials.gov. All patients provided written informed consent prior to study inclusion.

### 2.5 Statistical considerations

Continuous variables are presented as mean ± standard deviation (SD), and categorical variables as counts and percentages. Results of the 6WT are reported both as raw 6WD (meters ± SD) and as standardized z-scores adjusted for age and sex using population-based reference values. Objective functional impairment (OFI) was categorized based on z-scores using a four-grade scale: no OFI (z > −1), mild OFI (−1 to −1.9), moderate OFI (−2 to −2.9), and severe OFI (≤−3) (Tosic et al., 2020). Z-scores reflect the number of SDs by which an individual deviates from a spine-healthy reference population. (Tosic et al., 2020).^,^ (Gautschi et al., 2016b)

Changes between baseline and follow-up time points were analyzed using paired-sample t-tests. MCIDs were defined in accordance with previous literature as 1.6 points for VAS leg pain, 56 m for 6WD, and 0.6 for 6WT z-scores (Ziga et al., 2025).

Contingency table analyses were performed to evaluate sensitivity, specificity, positive predictive value (PPV), and negative predictive value (NPV) of achieving MCID at 1W for predicting MCID attainment at 1M. Consistency of MCID achievement across time points was assessed using Cochran's Q test (p > 0.05 indicating consistency). Agreement was evaluated using Fleiss' kappa, interpreted according to established benchmarks. Scores from 0.81 to 1.00 were classified as “almost perfect”, scores from 0.61 to 0.80 as “substantial”, scores from 0.41 to 0.60 as “moderate”, scores from 0.21 to 0.40 as “fair”, scores from 0.00 to 0.20 as “slight”, and scores of 0.00 or less as “poor” agreement (Landis and Koch, 1977).

All analyses were conducted using IBM SPSS Statistics version 27.0.0.0 (IBM Corp., Armonk, NY, USA). Statistical significance was set at p < 0.05.

## 3 Results

### 3.1 Patients’ demographics

A total of 45 consecutive patients undergoing ESI for sciatica due to DLD were included. The mean age was 51 years (SD 13). Detailed demographic and clinical characteristics are presented in Table 1.

**Table 1:** Patients demographics. Data is presented in count (%) or mean (standard deviation). ASA = American Society of Anaesthesiologists risk scale; BMI = body mass index,^a^ according to the British Medical Research Council (BMRC) paresis grading.

	Study group
**Age in years**	51 (13)
**Sex**	
Male	21 (47%)
Female	24 (53%)
**Body dimensions**	
Height in cm	170 (16.7)
Weight in kg	76 (21.5)
BMI in kg/m^2^	24.7 (4.1)
**Working status**	
Full-time	33 (74%)
Part-time	2 (4%)
Retired	2 (4%)
Disabled	8 (18%)
**Smoking status**	
Smoker	5 (11%)
Non-smoker	40 (89%)
**ASA risk scale**	
1	40 (89%)
2	4 (9%)
3	1 (2%)
**Lower Extremity Motor Deficit^a^**	
M1	0 (0%)
M2	0 (0%)
M3	0 (0%)
M4	4 (9%)
M5	41 (91%)
**Indication**	
Lumbar disc herniation	30 (67%)
Lateral recess stenosis	15 (33%)
**Type of steroid injection**	
Interlaminar epidural	2 (4%)
Transforaminal epidural	43 (96%)
**Affected Segment**	
L1/2	1 (2%)
L2/3	3 (7%)
L3/4	7 (15%)
L4/5	24 (53%)
L5/S1	10 (23%)

### 3.2 Change in subjective (VAS leg and VAS back) and objective outcome measures (6WT) from baseline to 1 month after epidural steroid infiltration

Table 2 shows the mean scores (SD) for VAS Leg, VAS Back and the 6WT from baseline until 1M postinterventional.

**Table 2:** Preoperative, 1 day- (1D), 1 week- (1W) and 1 month (1M) postinterventional subjective and objective outcome measures with change scores showed as mean [standard deviation]. 6WD = 6-min walking distance, VAS = visual analogue scale.

Measure	baseline	1D	1W	1M	Δ 1D -baseline (p-value)	Δ 1W - baseline (p-value)	Δ 1M - baseline (p-value)
**6WD (m)**	457 [92.4]	523 [70.6]	501 [66]	507 [89.7]	66 [83] **(p<0.001)**	44 [67.9] **(p<0.001)**	50 [80.1] **(p<0.001)**
**z-scores**	−1.0 [1.1]	−0.5 [1.1]	−0.49 [1.0]	−0.7 [1.2]	0.5 [1.6] **(p=0.02)**	0.51 [0.9] **(p<0.001)**	0.3 [1.1] **(p=0.02)**
**VAS Back**	2.3 [2.9]	0.9 [1.2]	0.8 [1.1]	0.7 [1.2]	−1.4 [1.9] **(p<0.001)**	−1.5 [2.1] **(p<0.001)**	−1.6 [2.4] **(p<0.001)**
**VAS Leg**	5.9 [1.8]	2.3 [1.1]	2.2 [1.4]	2.1 [1.5]	−3.6 [1.9] **(p<0.001)**	−3.7 [1.8] **(p<0.001)**	−3.8 [2.1] **(p<0.001)**

Following epidural steroid injection (ESI), a significant immediate improvement was observed across all measured outcomes. The mean 6WD improved from 457m (SD 92; z-score: −1.0, SD 1.1) at baseline to 523m (SD 71; z-score: −0.5, SD 1.1; p < 0.001) at the 1-day follow-up. Similarly, significant reductions in pain were reported from baseline to the 1-day follow-up, with a mean change of 2.3 (SD 1.1) in VAS Leg and 0.9 (SD 1.2) in VAS Back (all p < 0.001).

**Fig. 1:**
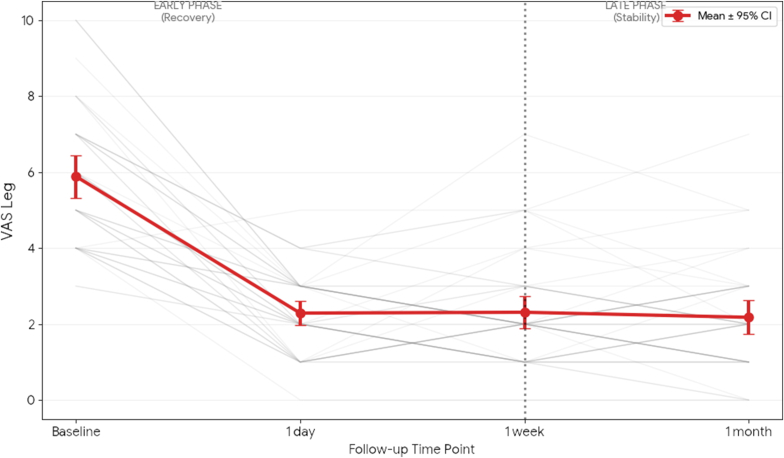
**Longitudinal Trajectories of Leg Pain.** Individual patient trajectories (grey lines, N = 45) and mean cohort trajectory (bold red line) for VAS Leg. Error bars represent the 95% Confidence Interval (CI). The vertical dashed line demarcates the early recovery phase from the late stability phase.

**Fig. 2:**
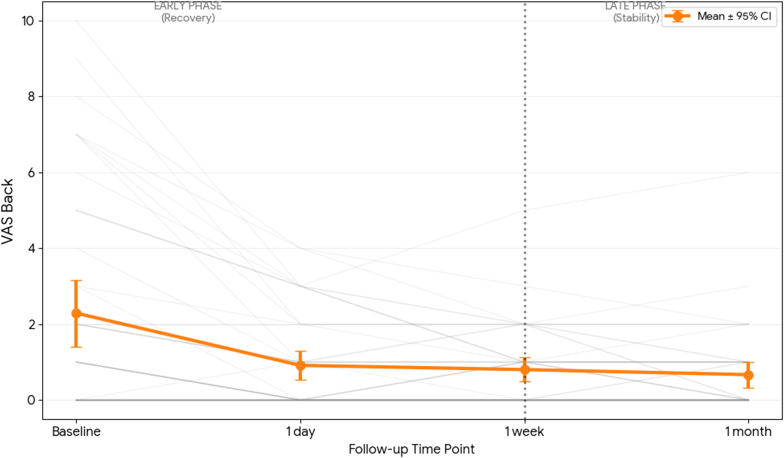
**Longitudinal Trajectories of Back Pain.** Individual patient trajectories (grey lines, N = 45) and mean cohort trajectory (bold orange line) for VAS Back. Error bars represent the 95% CI. The vertical dashed line demarcates the early recovery phase from the late stability phase.

**Fig. 3:**
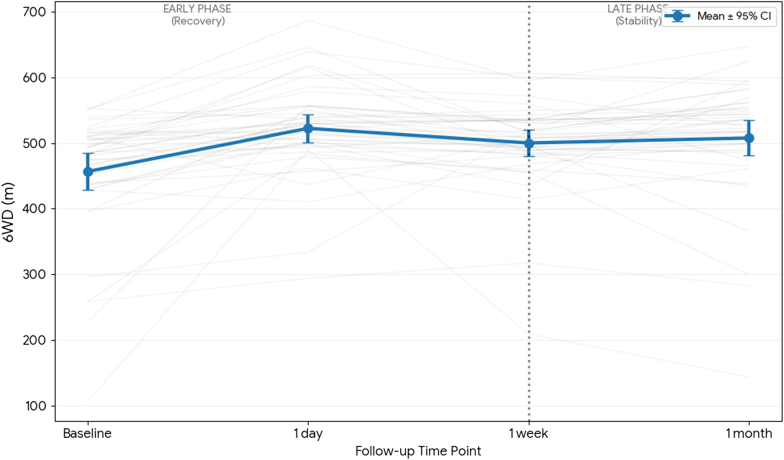
**Longitudinal Trajectories of Objective Physical Capacity.** Individual patient trajectories (grey lines, N = 45) and mean cohort trajectory (bold blue line) for the 6-min walking distance, 6WD (m). Error bars represent the 95% CI. The objective improvement in functional capacity demonstrates a clear plateau following the 1-week assessment.

Crucially, no significant improvement was observed after the 1-day follow-up for either subjective (VAS Leg, VAS Back) or objective outcomes (6WD, z-score). This longitudinal pattern, characterized by rapid early recovery followed by a maintenance phase, is illustrated in Fig. 1, Fig. 2, Fig. 3. The mean VAS Leg score (Fig. 1) and 6WD (Fig. 3) demonstrate a clear statistical plateau between 1 week and 1 month, where the 95% confidence intervals overlap almost entirely. Individual patient trajectories (grey lines) confirm that the therapeutic effect established within the first week remains stable through the mid-term follow-up.

### 3.3 Consistency and agreement of MCID detection

MCID achievement was consistent across postoperative time points for all outcome measures (Cochran's Q, all p > 0.05). Agreement of MCID classification across time was substantial for 6WD (Fleiss' κ = 0.67) and for z-scores (Fleiss' κ = 0.62). VAS Leg showed almost perfect MCID classification across time with Fleiss' κ = 0.82. Effect sizes ranged from poor to almost perfect, consistent with previous classifications (Landis and Koch, 1977).

### 3.4 Predictive value of early MCID achievement

Contingency table analyses demonstrated that patients achieving MCID at 1W were highly likely to also achieve MCID at 1M. Sensitivity and PPV at 1W were 100% (95% CI: 91 % - 100%) and 91% (95% CI: 78% - 98%) for VAS leg pain and 80% (95% CI: 65% - 99%) and 70% (95% CI: 47% - 87%) for raw 6WD. Specificity and NPV were 20% (95% CI: 1% - 72%) and 100% (95% CI: 3% - 100%) for VAS leg pain and 74% (95% CI: 54% - 89%) and 84% (95% CI: 71% - 99%) for raw 6WD. Detailed results are provided in Table 3, Table 4.

**Table 3:** Contingency table determining the specificity, sensitivity, negative predictive value (NPV) and positive predictive value (PPV) for subjective outcome measure – VAS Leg. Change score (MCID) of >1.6 for VAS Leg determined the responder to treatment. MCID = minimum clinically important difference, VAS = visual analogue scale, CI – confidence interval.

1 day vs 1 month								
VAS Leg		MCID 1M		Total	Sensitivity (95% CI)	Specificity (95% CI)	PPV (95% CI)	NPV (95% CI)
		responder	non- responder					
**MCID 1D**	**responder**	36	3	39	90% (76% - 97%)	40% (5% - 85%)	92% (79% - 98%)	33% (4% - 78%)
	**non- responder**	4	2	6				
**Total**		40	5	45				

1 week vs 1 month								
VAS Leg		MCID 1M		Total	Sensitivity (95% CI)	Specificity (95% CI)	PPV (95% CI)	NPV (95% CI)
		responder	non- responder					
**MCID 1W**	**responder**	40	4	44	100% (91 % - 100%)	20% (1% - 72%)	91% (78% - 98%)	100% (3% - 100%)
	**non- responder**	0	1	1				
**Total**		40	5	45

**Table 4:** Contingency table determining the specificity, sensitivity, negative predictive value (NPV) and positive predictive value (PPV) for objective outcome measure – raw 6WD. Change score (MCID) of >56m for raw 6WD determined the responder to treatment. MCID = minimum clinically important difference, 6WD = 6-min walking distance, CI – confidence interval.

1 day vs 1 month								
Raw 6WD		MCID 1M		Total	Sensitivity (95% CI)	Specificity (95% CI)	PPV (95% CI)	NPV (95% CI)
		responder	non- responder					
**MCID 1D**	**responder**	13	7	20	72% (56% - 94%)	74% (39% - 79%)	65% (41% - 80%)	80% (54% - 94%)
	**non- responder**	5	20	25				
**Total**		18	27	45				

1 week vs 1 month								
Raw 6WD		MCID 1M		Total	Sensitivity (95% CI)	Specificity (95% CI)	PPV (95% CI)	NPV (95% CI)
		responder	non- responder					
**MCID 1W**	**responder**	14	7	21	80% (65% - 99%)	74% (54% - 89%)	70% (47% - 87%)	84% (71% - 99%)
	**non- responder**	4	20	24				
**Total**		18	27	45				

## 4 Discussion

This study provides a longitudinal evaluation of the 6WT in conservatively treated patients with sciatica due to DLD undergoing ESI. Specifically, we assessed its ability to capture temporal changes in physical capacity, discriminate between responders and non-responders, and predict mid-term treatment success based on early postinterventional outcomes. While prior investigations have largely focused on surgical cohorts, evidence for conservatively managed patients—who represent the majority of individuals with DLD—remains limited (Sosnova et al., 2020; Zeitlberger et al., 2020; Ziga et al., 2023, 2024, 2025; Maldaner et al., 2020a, 2020b).

Three principal findings emerged. Firstly, the 6WT reliably quantified functional impairment at all assessed time points and sensitively captured treatment-related changes. Its integration with PROMs, such as VAS leg and back pain, enabled a more comprehensive evaluation of patient outcomes. (Stienen et al., 2019b)^,^ (Maldaner et al., 2020a)^,^ (Ziga et al., 2024) Secondly, both subjective well-being and objective physical performance peaked at 1D following ESI, with no significant further improvement observed thereafter. Thirdly, achievement of MCID within 1W reliably identified patients who would also achieve MCID at 1M, demonstrating the predictive value of early functional assessment using the 6WT.

### 4.1 Temporal changes in PROMs and objective function

Subjective PROMs are well-established tools used to assess patients' perceived functional impairment (Mannion et al., 2005, 2015). Previous studies on surgically treated patients indicate that the most significant improvements in PROMs occur shortly after surgery (Ziga et al., 2023; Whitmore et al., 2015). For instance, in Whitmore's cohort of 148 patients, the one-year outcome could be reliably estimated based on the three-month results, as PROMs showed little change after this period (Whitmore et al., 2015). This finding aligns with our study on conservatively treated patients, where PROM results plateaued very early, at the 1D FU. In contrast to surgical cohorts, where 6WT results continued to improve and surpassed PROMs at the 1M FU, our study found that objective outcomes measured by the 6WT in patients treated with ESI also plateaued at the 1D FU. This suggests no further improvement in both - objective and subjective outcome measures, beyond this point in actual cohort.

Despite the absence of additional improvement beyond day 1 at the group level, we deliberately evaluated patients' outcome at both 1D and 1W after ESI. This analytical decision was based on pharmacological and pathophysiological considerations. At the 1D FU, outcome measures may still be substantially influenced by the short-term analgesic effects of the local anesthetic component of the injection. In contrast, assessments at 1W are less likely to be confounded by residual anesthetic effects and are therefore more reflective of the anti-inflammatory action of corticosteroids, which is considered the primary driver of sustained clinical benefit after ESI. Accordingly, evaluating patients' outcome at 1W provides a clinically more meaningful estimate of treatment response attributable to corticosteroid efficacy, while the 1D assessment offers insight into early functional changes under transient analgesia.

### 4.2 Minimum clinically important difference and outcome prediction

The results of this study suggest that the clinical effectiveness of ESI is established much earlier than the traditional 1 month FU. As seen in the individual trajectory analyses (Fig. 1, Fig. 2, Fig. 3), the 'Late Phase' (1W to 1M) is defined by a stabilization of both subjective pain and objective functional capacity. This plateau suggests that a 1W assessment captures the near-maximal treatment effect, providing a reliable early window to predict the mid-term outcome and guide clinical decision-making.

Our analysis explored whether achieving the MCID within 1W can predict responders to treatment at the 1M FU. Both subjective and objective outcomes presented with high sensitivity (VAS Leg: 100% [95% CI: 91 % - 100%], 6WD: 80% [95% CI: 65% - 99%]) and high PPV (VAS Leg: 91% [95% CI: 78% - 98%], 6WD: 70% [95% CI: 47% - 87%]). While the 1-week VAS Leg MCID serves as an excellent screening tool for identifying potential 1-month responders (sensitivity: 100%, 95% CI: 91% - 100%), its low specificity (20%, 95% CI: 1% - 72%) suggests it cannot reliably identify non-responders. Therefore, clinicians should view early VAS improvement as an indicator of likely success rather than a definitive predictor of overall outcome. Notably, the 6WD also demonstrated high specificity (74% [95% CI: 54% - 89%]) and high NPV (84% [95% CI: 71% - 99%]), indicating that the 6WT is able to effectively and objectively determine responders within 1W post-ESI. These findings are significant for spine surgeons in counseling patients in day-to-day praxis and have important implications for physicians who aim to set and communicate realistic expectations for future improvements.

Beyond absolute changes, our analysis of MCID consistency and agreement provides important methodological insight. MCID achievement was statistically consistent across all follow-up time points, indicating that the proportion of patients classified as responders remained stable over time. Agreement of MCID classification ranged from substantial to almost perfect, with particularly high agreement observed for VAS Leg and 6WD. This observed high agreement is clinically relevant, as it indicates that early classification as a responder or non-responder is unlikely to be reversed at later follow-up.

From a clinical perspective, these results suggest that routine assessment within the first week following ESI is sufficient to estimate mid-term treatment effectiveness. The 6WT, as an objective, patient-performed, and valid measure of physical capacity, complements traditional PROMs and mitigates some of their inherent subjectivity and ceiling effects. Incorporating objective functional testing into early follow-up may therefore enhance counseling, expectation management, and timely consideration of alternative treatment strategies in non-responders.

### 4.3 Strengths and limitations

The strengths of this study include its prospective design and predefined protocol, which incorporated both widely used subjective PROMs (VAS Leg and Back) and the validated 6WT for physical capacity (Maldaner et al., 2020a). By restricting inclusion to patients with sciatica, our findings are more directly applicable to this highly prevalent and clinically distinct subgroup, avoiding confounding from heterogeneous pathologies and treatment strategies.

Nevertheless, certain limitations warrant consideration. First, the restriction to smartphone users introduces potential socioeconomic bias and limits generalizability. Patients’ preference may change with the applied objective and subjective outcome measures as well as with the education and socioeconomic background of the patient. Our study is therefore limited to the applied measures and cannot simply be generalized to patients who do not, or cannot use the certain smartphone technology. Future studies might address this “digital divide” by giving patients the opportunity to assess the physical capacity using the same e.g. hospitals technological resources. Second, the 1-day assessment likely reflects the immediate pharmacological effect of the local anesthetic (Rapidocain) rather than the long-term anti-inflammatory effect of the corticosteroid (Dexamethasone). Furthermore, without a sham-controlled group, we cannot definitively exclude the impact of natural history or regression to the mean on these early trajectories. Third, we could not account for geographic, climatic, or seasonal variations that may affect the walking distance. Forth, our sample size limited further subgroup analyses. However, future investigations focusing on larger subgroups may help elucidate potential functional disparities between different pathologies.

## 5 Conclusion

In patients with sciatica due to DLD undergoing ESI, the 6WT performed within one week is associated with treatment success at one month, supporting its role as a valuable objective tool in early outcome assessment.

## Ethical approval

All procedures performed in studies involving human participants were in accordance with the ethical standards of the institutional and/or national research committee and with the 1964 Helsinki declaration and its later amendments or comparable ethical standards.

## Declaration of generative AI and AI-assisted technologies in the manuscript preparation process

During the preparation of this work the authors used ChatGPT in order to improve the readability and language of the manuscript. After using this tool/service, the authors reviewed and edited the content as needed and take full responsibility for the content of the published article.

## Declaration of competing interest

The authors declare that they have no known competing financial interests or personal relationships that could have appeared to influence the work reported in this paper.
