# Supplementary material for: Early assessment of physical capacity and pain is associated with 1-month response following epidural steroid injection in patients with sciatica due to degenerative lumbar disorders
Source: Brain Spine. 2026 May 29;6:106111. doi: 10.1016/j.bas.2026.106111 (PMC13241758; doi:10.1016/j.bas.2026.106111)
Supplement: Multimedia component 1 [file mmc1.docx]

**Supplemental Digital Content 1.**

**1. Inclusion and Exclusion Criteria for the study cohort**

Patients fulfilling all of the inclusion criteria were considered for this study:

• Patients with DLD not primarily treated surgically but scheduled for elective epidural or transforaminal steroid injection in an outpatient setting with (1) lumbar disc herniation (LDH) or (2) lumbar spinal stenosis (LSS)

• Male and Female subjects ≥ 18 years

• Written informed consent.

Patients were not enrolled if any of the following exclusion criteria were met:

- Pregnancy
- Inability to walk (extreme pain or severe neurological deficits)
- Severe heart failure corresponding to > NYHA III
- Lung diseases (e.g. lung cancer, diffuse parenchymal lung disease, severe chronic obstructive lung disease (COPD) corresponding to > Gold III)
- Other medical reasons interfering with the patient’s ability to walk and perform the 6WT (e.g. osteoarthritis disease of the lower extremities, Parkinson’s disease, hip or knee prosthesis, peripheral artery disease causing intermittent claudication, etc.)
- Inability to complete assessment (planning to move, no smartphone, etc.)
